# Simultaneous Patch-Group Sparse Coding with Dual-Weighted *ℓ*_p_ Minimization for Image Restoration

**DOI:** 10.3390/mi12101205

**Published:** 2021-10-01

**Authors:** Jiachao Zhang, Ying Tong, Liangbao Jiao

**Affiliations:** 1 Artificial Intelligence Industrial Technology Research Institute, Nanjing Institute of Technology, Nanjing 211167, China; jiaoliangbao@njit.edu.cn; 2 Jiangsu Engineering Research Center of IntelliSense Technology and System, Nanjing Institute of Technology, Nanjing 211167, China

**Keywords:** image restoration, sparse coding, SPG-SC, dual-weighted *ℓ_p_* minimization, nonlocal sparse representation, ADMM

## Abstract

Sparse coding (SC) models have been proven as powerful tools applied in image restoration tasks, such as patch sparse coding (PSC) and group sparse coding (GSC). However, these two kinds of SC models have their respective drawbacks. PSC tends to generate visually annoying blocking artifacts, while GSC models usually produce over-smooth effects. Moreover, conventional $\ell_{1}$ minimization-based convex regularization was usually employed as a standard scheme for estimating sparse signals, but it cannot achieve an accurate sparse solution under many realistic situations. In this paper, we propose a novel approach for image restoration via simultaneous patch-group sparse coding (SPG-SC) with dual-weighted $\ell_{p}$ minimization. Specifically, in contrast to existing SC-based methods, the proposed SPG-SC conducts the local sparsity and nonlocal sparse representation simultaneously. A dual-weighted $\ell_{p}$ minimization-based non-convex regularization is proposed to improve the sparse representation capability of the proposed SPG-SC. To make the optimization tractable, a non-convex generalized iteration shrinkage algorithm based on the alternating direction method of multipliers (ADMM) framework is developed to solve the proposed SPG-SC model. Extensive experimental results on two image restoration tasks, including image inpainting and image deblurring, demonstrate that the proposed SPG-SC outperforms many state-of-the-art algorithms in terms of both objective and perceptual quality.

## 1. Introduction

As an important task in the field of image processing, image restoration has attracted considerable interests for many researchers and been widely applied in various areas such as medical image analysis [1], remote sensing [2] and digital photography [3]. The goal of image restoration is to reconstruct a high-quality image from its degraded (e.g., noisy, blurred or pixels missing) observation, which is typically an ill-posed inverse problem and can be mathematically modeled as

(1) y=Hx+n,

where x, y are lexicographically stacked representations of the original image and degradation observation, respectively. H stands for a non-invertible degradation matrix and n is usually assumed to be an additive Gaussian white noise. Through selecting specific values for H, the model in Equation (1) can represent different image restoration tasks. For instance, when H is an identity matrix, Equation (1) represents a simple image denoising problem [4,5]; when H is a blur matrix, it is an image deblurring problem [6,7]; when H is a diagonal matrix whose diagonal entries are either 1 or 0 (keeping or killing corresponding pixels), it denotes an image inpainting problem [8,9], and when H is a random projection matrix, it is an image compressive sensing recovery problem [10,11]. In this paper, we mainly focus on image inpainting and image deblurring problems.

Due to the ill-posed nature of image restoration, image prior of the original image x is usually employed to regularize the solution space and finally achieve a high-quality reconstructed image. In general, image prior-based regularization for image restoration can be expressed by the following minimization problem,

(2) $$\hat{x}=\arg\min_{x}\frac{1}{2}{∥y-Hx∥}_{2}^{2}+\lambda \;\Phi \left(x\right),$$

where ${∥\cdot ∥}_{2}$ denotes the $\ell_{2}$-norm and the first term in Equation (2) is the fidelity term. Φ(x) represents the regularization term, which takes the employed image priors into account. λ is a regularization parameter to balance these two terms. To tackle the ill-posed image restoration problem, image prior knowledge plays a critical role in enhancing the performance of image restoration algorithms. In other words, it is vital to devise an effective regularization model of Equation (2) for image restoration, which reflects the image prior information. During recent decades, various image prior-based regularization models have been proposed in the literature to depict the statistical features of natural images [4,5,6,7,8,9,10,11,12,13,14,15,16,17,18,19,20,21,22].

Early regularization models mainly consider the prior on the level of pixels, such as total variation (TV) [13,14], which actually assumes that the nature image gradient obeys Laplacian distribution. TV-based methods remove the noise artifacts effectively, but they often erase the fine details and lean to over-smooth the images due to the piecewise constant assumption [6,7].

Another crucial property of natural images is to model the prior on image patches. One representative research is sparse coding (SC) model, which is generally classified into two categories: patch sparse coding (PSC) [15,16,17,23,24] and group sparse coding (GSC) [5,10,11,18,19,20,21,25,26,27]. PSC usually assumes that each patch of an image can be accurately represented by a sparse coefficient vector whose entries are mostly zeros or close to zero based on a dictionary of atoms. A series of dictionary learning approaches have been proposed and exploited to image restoration and other image processing tasks [17,28,29,30]. For example, the famous dictionary learning method is KSVD [1], which has achieved promising performance in numerous applications, ranging from image denoising to computer vision [31,32]. Mairal et al. [28] proposed an online dictionary learning (ODL) approach to various machine learning and image processing tasks. However, in image restoration, PSC is usually unstable and tends to generate visual annoying blocking artifacts [33]. Moreover, the PSC model not only requires expensive computation to learn an off-the-shelf dictionary, but also commonly takes no account of the correlation of similar patches [10,11,18,19].

To overcome the above-mentioned disadvantages of PSC, recent advances of GSC, which inspired by the success of nonlocal self-similarity (NSS) prior in images [4], exploit nonlocal similar *patch group* as the basic unit for SC and have shown great potential in a variety of image restoration tasks [5,10,11,18,19,21,27,34,35,36]. For instance, a very popular method is BM3D [5], which exploited the NSS prior to construct 3D arrays and conducted these 3D arrays with transform domain filtering. To the best of our knowledge, it is the earliest one that uses both NSS prior and sparsity for image restoration. Mairal et al. [18] advanced the idea of NSS by GSC. LPG-PCA [35] calculated statistical parameters for PCA learning through using the nonlocal similar patches as data samples. Dong et al. [36] proposed a joint local and nonlocal sparsity constraints for image restoration. Zhang et al. [10] exploited the NSS prior for image restoration under the framework of group-based sparse representation. Since the matrix formed by nonlocal similar patches in a natural image is of low-rank, in [37,38,39,40,41,42,43], the authors transformed the image restoration into the low-rank matrix approximation problems, which have achieved highly competitive reconstruction results. Though the GSC model has achieved a great success in miscellaneous image restoration applications, it tends to smooth out some fine details of the reconstructed images [43]. Furthermore, SC-based image restoration problem is naturally modeled using the $\ell_{0}$-norm penalty [44]. However, the fact of $\ell_{0}$ minimization being NP-hard has encouraged us to relax the $\ell_{0}$ minimization to some tractable alternatives. A widely used scheme is that $\ell_{0}$ minimization is replaced by its convex $\ell_{1}$ counterpart, which is widely used as a standard scheme for estimating sparse signals, and many optimization algorithms have been developed to solve the $\ell_{1}$ minimization problem [45,46,47]. However, a fact that cannot be ignored is, $\ell_{1}$ minimization cannot achieve an accurate sparsity solution under many practical situations including image restoration problems [10,11,19,48]. Moreover, weighted $\ell_{1}$ minimization [49], $\ell_{p}$ [50] minimization, and even the weighted $\ell_{p}$ [51] minimization which has been demonstrated achieving better sparse solutions, are proposed to estimate sparse signals [11] for further practical use.

Bearing the above concerns in mind, this paper proposes a novel approach for image restoration via simultaneous patch-group sparse coding (SPG-SC) with a dual-weighted $\ell_{p}$ minimization. The local and nonlocal sparse representations synchronously exploited in SPG-SC could eliminate the block artifacts and over-smooth often occurred in PSC or GSC-based methods. Moreover, a new dual-weighted $\ell_{p}$ minimization based on non-convex regularization is presented, which aims for enhancing the sparse representation capability of the proposed SPG-SC framework in image restoration tasks. The major contributions of this paper are summarized as follows. First, compared to the existing SC-based methods, the proposed SPG-SC exploits the local sparsity and nonlocal sparse representation simultaneously. Secondly, to improve the sparse representation capability of the proposed SPG-SC model, a dual-weighted $\ell_{p}$ minimization-based non-convex regularization is proposed. Thirdly, to make the optimization tractable, we develop a non-convex generalized iteration shrinkage algorithm based on the alternating direction method of multipliers (ADMM) framework to solve the proposed SPG-SC model. Experimental results demonstrate that in the tasks of image inpainting and image deblurring, our proposed SPG-SC outperforms many state-of-the-art methods both quantitatively and qualitatively.

The remainder of this paper is organized as follows. Section 2 introduces the related works about sparse coding for image processing. Section 3 presents a novel sparse coding model, i.e., SPG-SC for image restoration. Section 4 describes the experimental results for image inpainting and image deblurring. Finally, concluding remarks are provided in Section 5.

## 2. Sparse Coding for Image Processing

### 2.1. Patch Sparse Coding

SC exhibits promising performance for various image processing tasks [15,16,17,23], which assumes that an image can be spanned by a set of bases or dictionary atoms in a transfer domain. According to [15], the basic unit of SC for images is patch. Mathematically, denote an image by $x\in R^{N}$; let $x_{i}=R_{i}x,i=1,\ldots ,n$, denote an image patch of size $\sqrt{b}\times \sqrt{b}$ extracted from location *i*, where $R_{i}$ represents the matrix extracting the patch $x_{i}$ from x. For a given dictionary $D\in R^{b\times M}$, b≤M, the sparse coding processing of each patch $x_{i}$ is to obtain a sparse vector $\alpha_{i}$ such that $x_{i}\approx D\alpha_{i}$. Please note that most of the elements are zeros in vector $\alpha_{i}$. In general, the sparse coding problem of $x_{i}$ over D is solved by the following optimization problem,

(3) $${\hat{\alpha }}_{i}=\arg\min_{\alpha_{i}}\left(\frac{1}{2}{∥x_{i}-D\alpha_{i}∥}_{2}^{2}+\lambda {∥\alpha_{i}∥}_{0}\right),$$

where λ is a non-negative parameter to balance the fidelity and the sparsity regularization; the notation ${∥\cdot ∥}_{0}$ is the $\ell_{0}$-norm (quasi-norm), i.e., counting the number of nonzero elements in vector $\alpha_{i}$. Following this, the whole image x can be sparsely represented by a set of sparse codes ${\left\{\alpha_{i}\right\}}_{i=1}^{n}$. Concatenating *n* patches, let $X=\left[x_{1},\ldots ,x_{n}\right]\in R^{b\times n}$ denote all the patches extracted from the image. Since D is shared by these patches, we thus have

(4) $$\hat{A}=\arg\min_{A}\left(\frac{1}{2}{∥X-DA∥}_{F}^{2}+\lambda {∥A∥}_{0}\right),$$

where ${∥\cdot ∥}_{F}$ represents the Frobenius norm, $A=\left[\alpha_{1},\ldots ,\alpha_{n}\right]\in R^{M\times n}$ is the sparse coefficient matrix, and the $\ell_{0}$-norm is imposed on each column of A (corresponding to each patch).

### 2.2. Group Sparse Coding

Instead of using a single patch as the basic unit in PSC, GSC employs *patch group* as its unit. In this subsection, we briefly introduce the GSC model [10,11,18,19,52]. Specifically, for an image x, we first divide it into *n* overlapped patches $x_{i}$ of size $\sqrt{b}\times \sqrt{b},i=1,\ldots ,n$. Secondly, in contrast to PSC, for each patch $x_{i}$, we search *m* patches that are the most similar to itself within an L×L sized window to form a *patch group* $X_{G_{i}}$, denoted by $X_{G_{i}}=\left\{x_{i,1},\ldots ,x_{i,m}\right\}$, where $x_{i,m}$ denotes the *m*-th similar patch (column vector) in the *i*-th *patch group*. It is worth noting that the K-Nearest Neighbor (KNN) algorithm [53] is used to search similar patches here. Finally, similar to PSC, given a dictionary $D_{G_{i}}\in R^{b\times K}$, each group $X_{G_{i}}$ can be sparsely represented $B_{G_{i}}={D_{G_{i}}}^{T}X_{G_{i}}$ and can be solved by the following $\ell_{0}$ minimization problem,

(5) $${\hat{B}}_{G_{i}}=\arg\min_{B_{G_{i}}}\left(\frac{1}{2}∥X_{G_{i}}-D_{G_{i}}B_{G_{i}}{∥}_{F}^{2}+\lambda {∥B_{G_{i}}∥}_{0}\right),$$

where $B_{G_{i}}$ is the group sparse coefficient of each group $X_{G_{i}}$, and the $\ell_{0}$-norm is imposed on each column in $B_{G_{i}}$. To put all groups in one shot, we define the notation $Q_{i}\in R^{n\times m}$, which is the searching and extracting operations of the similar patches for the *i*-th patch, i.e., $X_{G_{i}}=XQ_{i}$. Concatenating *n* patch groups, we thus have

(6) $$X_{G}=X\left[Q_{1},\ldots ,Q_{n}\right]=XQ\in R^{b\times (mn)}.$$

Since each patch group has its own dictionary in GSC and they are not necessarily shared, let

(7) $$\begin{array}{ccc} D_{G} & = & \left[D_{G_{1}},\ldots ,D_{G_{n}}\right]\in R^{b\times (nK)}, \end{array}$$

(8) $$\begin{array}{ccc} {\bar{B}}_{G} & = & \left[{\bar{B}}_{G_{1}},\ldots ,{\bar{B}}_{G_{n}}\right]\in R^{\left(nK)\times (mn\right)}, \end{array}$$

where ${\left\{{\bar{B}}_{G_{i}}\right\}}_{i=1}^{n}\in R^{nK\times m}$ is an expanded (longer with more rows) version of $B_{G_{i}}\in R^{K\times m}$, with $B_{G_{i}}$ in the corresponding locations (from ((i−1)K+1)-th row to (iK)-th row) but zeros elsewhere, i.e., corresponding to $D_{G_{i}}$ in $D_{G}$. The GSC problem we are going to solve now becomes

(9) $${\hat{\bar{B}}}_{G}=\arg\min_{{\bar{B}}_{G}}\left(\frac{1}{2}∥X_{G}-D_{G}{\bar{B}}_{G}{∥}_{F}^{2}+\lambda {∥{\bar{B}}_{G}∥}_{0}\right),$$

where the $\ell_{0}$-norm is again imposed on each column, and this holds true for the following derivations in this paper. Please note that both X in PSC and $X_{G}$ in GSC are constructed from the same original image x.

## 3. Image Restoration Using Simultaneous Patch-Group Sparse Coding with Dual-Weighted $\ell_{p}$ Minimization

As mentioned before, the PSC model usually leads to the visual annoying blocking artifacts, while the GSC model is apt to produce the over-smooth effect in various image restoration tasks. In this section, to cope with these problems, we propose a novel simultaneous patch-group sparse coding (SPG-SC) model for image restoration, rather than using the PSC model in Equation (4) or the GSC model in Equation (9) individually.

Before doing this, we first make some preliminary transformations to connect the PSC model in Equation (4) with the GSC model in Equation (9). Specifically, recall that each patch (column) in the patch-group $X_{G}$ is from X and it can be sparsely represented by Equation (4). Hence, except for the sparse coding processing in Equation (9), we can also have

(10) $$X_{G}=DA_{G},$$

where $A_{G}\in R^{M\times (mn)}$ is composed of the corresponding columns in A, namely $A_{G}$ is an expanded version of A in Equation (4), where each column is reproduced by *m* times according to the patch searching in $X_{G}$. In this case, similar to Equation (3), $A_{G}$ can be solved by

(11) $${\hat{A}}_{G}=\arg\min_{A_{G}}\left(\frac{1}{2}{∥X_{G}-DA_{G}∥}_{F}^{2}+\lambda {∥A_{G}∥}_{0}\right).$$

### 3.1. Modeling of Simultaneous Patch-Group Sparse Coding for Image Restoration

Now, after integrating the above PSC model in Equation (11) and the GSC model in Equation (9) into the regularization-based framework of Equation (2), the proposed SPG-SC model for image restoration can be represented as follows:

(12) $$\begin{array}{cc} \left({\hat{X}}_{G},{\hat{A}}_{G},{\hat{\bar{B}}}_{G}\right) & =\underset{X_{G},A_{G},{\bar{B}}_{G}}{\mathrm{argmin}}\frac{1}{2}{∥Y_{G}-H_{G}X_{G}∥}_{F}^{2}\\ \; & \;\;+\frac{\mu_{1}}{2}{∥X_{G}-DA_{G}∥}_{F}^{2}+\lambda {∥A_{G}∥}_{0}\\ \; & \;\;+\frac{\mu_{2}}{2}∥X_{G}-D_{G}{\bar{B}}_{G}{∥}_{F}^{2}+\rho {∥{\bar{B}}_{G}∥}_{0}, \end{array}$$

where ρ is now playing the same role of λ as in Equation (9). $Y_{G}$ is obtained from y in the same procedure of $X_{G}$, and similarly for $H_{G}$, which is obtained from H. Here we introduce the parameters $\mu_{1}$ and $\mu_{2}$ to make the solution of Equation (12) more feasible.

However, it is well-known that $\ell_{0}$ minimization is discontinuous and NP-hard, solving Equation (12) is a difficult combinatorial optimization problem. For this reason, $\ell_{0}$ minimization is usually relaxed by the $\ell_{1}$ minimization to make the optimization tractable. Unfortunately, for some practical cases, such as image restoration problems [10,11,19,48], $\ell_{1}$ minimization is just an estimation to the $\ell_{0}$ minimization and cannot obtain the desirable sparse solution. Therefore, inspired by Zha’s work [11], which has demonstrated that the weighted $\ell_{p}$ minimization can achieve a better sparse solution than some existing minimization methods such as $\ell_{1}$ minimization [49], $\ell_{p}$ minimization [50] and the weighted $\ell_{1}$ minimization [51], we propose a new dual-weighted $\ell_{p}$ minimization based on our proposed SPG-SC framework for image restoration. To be concrete, Equation (12) can be rewritten as

(13) $$\begin{array}{cc} \left({\hat{X}}_{G},{\hat{A}}_{G},{\hat{\bar{B}}}_{G}\right) & =\underset{X_{G},A_{G},{\bar{B}}_{G}}{\mathrm{argmin}}\frac{1}{2}{∥Y_{G}-H_{G}X_{G}∥}_{F}^{2}\\ \; & \;\;+\frac{\mu_{1}}{2}{∥X_{G}-DA_{G}∥}_{F}^{2}+\lambda {∥W_{G}\circ A_{G}∥}_{p}\\ \; & \;\;+\frac{\mu_{2}}{2}∥X_{G}-D_{G}{\bar{B}}_{G}{∥}_{F}^{2}+\rho {∥K_{G}\circ {\bar{B}}_{G}∥}_{p}, \end{array}$$

where ∘ is the element-wise product of two matrices (Hadamard product). The notation ${∥\cdot ∥}_{p}$ denotes the $\ell_{p}$-norm to characterize the sparsity of sparse coefficients. It is worth noting that we employ the *p*-norm to matrix here and $\ell_{p}$-norm is imposed to each column of sparse coefficients. $W_{G}$ and $K_{G}$ are the weights of sparse coefficient $A_{G}$ and $B_{G}$, respectively. The weights could improve the representation capability of the sparse coefficients [51].

### 3.2. Generalized Iteration Shrinkage Algorithm Based on the ADMM Framework to Solve the Proposed SPG-SC Model

Since the objective function of Equation (13) is a large scale non-convex optimization problem, and to make the optimization tractable, we adopt a generalized iteration shrinkage algorithm based on the ADMM framework [54,55] to solve it, which has demonstrated to be quite effective, making each sub-problem solved efficiently. Specifically, the Equation (13) can be translated into the following five iterative steps by exploiting ADMM scheme:

(14) $$\begin{array}{cc} {X_{G}}^{\left(t+1\right)} & =\arg\min_{X_{G}}\frac{1}{2}{∥Y_{G}-H_{G}X_{G}∥}_{F}^{2}\\ \; & \;\;+\frac{\mu_{1}}{2}{∥X_{G}-DA_{G}^{\left(t\right)}-C^{\left(t\right)}∥}_{F}^{2}\\ \; & \;\;+\frac{\mu_{2}}{2}{∥X_{G}-D_{G}{{\bar{B}}_{G}}^{\left(t\right)}-J^{\left(t\right)}∥}_{F}^{2}, \end{array}$$

(15) $$\begin{array}{cc} {A_{G}}^{\left(t+1\right)} & =\arg\min_{A_{G}}\lambda ∥W_{G}\circ A_{G}{∥}_{p}+\frac{\mu_{1}}{2}{∥X_{G}^{\left(t+1\right)}-DA_{G}-C^{\left(t\right)}∥}_{F}^{2}, \end{array}$$

(16) $$\begin{array}{cc} {{\bar{B}}_{G}}^{\left(t+1\right)} & =\arg\min_{{\bar{B}}_{G}}\rho ∥K_{G}\circ {\bar{B}}_{G}{∥}_{p}+\frac{\mu_{2}}{2}{∥X_{G}^{\left(t+1\right)}-D_{G}{\bar{B}}_{G}-J^{\left(t\right)}∥}_{F}^{2}, \end{array}$$

(17) $$\begin{array}{cc} C^{\left(t+1\right)} & =C^{\left(t\right)}-\left(X_{G}^{\left(t+1\right)}-DA_{G}^{\left(t+1\right)}\right), \end{array}$$

(18) $$\begin{array}{cc} J^{\left(t+1\right)} & =J^{\left(t\right)}-\left(X_{G}^{\left(t+1\right)}-D_{G}{{\bar{B}}_{G}}^{\left(t+1\right)}\right). \end{array}$$

Obviously, the minimization of Equation (13) falls into three sub-problems, i.e., $X_{G}$, $A_{G}$ and ${\bar{B}}_{G}$ sub-problem. Fortunately, there is an efficient solution to each sub-problem, which will be discussed in the following subsections. Moreover, each problem can be solved patch by patch for image restoration. To be concrete, take the *i*-th patch $x_{i}$ as an example, $y_{i}=H_{i}x_{i}$, where $H_{i}$ represents the degraded matrix in the *i*-th patch. Recall that $A_{G}$ is an expanded version of A in the PSC model and we have $x_{i}=D\alpha_{i}$. After the $\alpha_{i}$ is solved, we can straightforwardly obtain $A_{G}$. In the GSC model, let $\beta_{i}$ concatenate all the group coefficients including the *i*-th patch; we thus have $x_{i}=D_{G}\beta_{i}$. Then, we consider solving the problem for each patch and the superscript *t* is omitted for conciseness. More specifically, we translate the $A_{G}$ sub-problem to ${\left\{\alpha_{i}\right\}}_{i=1}^{n}$ sub-problem, and translate the ${\bar{B}}_{G}$ sub-problem to ${\left\{B_{G_{i}}\right\}}_{i=1}^{n}$ sub-problem, and translate the $X_{G}$ sub-problem to ${\left\{x_{i}\right\}}_{i=1}^{n}$ sub-problem, respectively.

#### 3.2.1. $X_{G}$ Sub-Problem

Given $A_{G}$ and ${\bar{B}}_{G}$, $X_{G}$ sub-problem in Equation (14) for each patch $x_{i}$, becomes

(19) $$\begin{array}{cc} {\hat{x}}_{i} & =\arg\min_{x_{i}}\frac{1}{2}∥y_{i}-H_{i}x_{i}{∥}_{2}^{2}+\frac{\mu_{1}}{2}{∥x_{i}-D\alpha_{i}-c_{i}∥}_{F}^{2}\\ \; & \;+\frac{\mu_{2}}{2}{∥x_{i}-D_{G}\beta_{i}-j_{i}∥}_{F}^{2}, \end{array}$$

It can be seen that Equation (19) is essentially a minimization problem of strictly convex quadratic function, and therefore, it admits a closed-form solution for $x_{i}$, which can be expressed as

(20) $$\begin{array}{cc} {\hat{x}}_{i} & ={\left({H_{i}}^{T}H_{i}+\left(\mu_{1}+\mu_{2}\right)I\right)}^{-1}\left({H_{i}}^{T}y_{i}+\mu_{1}\left(D\alpha_{i}+c_{i}\right)+\mu_{2}\left(D_{G}\beta_{i}+j_{i}\right)\right), \end{array}$$

where I is an identity matrix with the desired dimensions. $c_{i}$ and $j_{i}$ are the corresponding elements of C and J, respectively. It is worth noting that each $x_{i}$ is jointly estimated in Equation (20) using both PSC ($A_{G}$) in Equation (11) and GSC (${\bar{B}}_{G}$) in Equation (9) in one shot. In our experiments, we notice that this joint estimation plays a key role in the performance improvement of our proposed SPG-SC model for image restoration (see Section 4 for more details).

#### 3.2.2. $A_{G}$ Sub-Problem

As mentioned before, $A_{G}$ is an expanded version of A, and therefore $A_{G}$ can be solved by the A sub-problem. Based on Equation (15), for *i*-th patch, $\alpha_{i}$ sub-problem can be rewritten as

(21) $${\hat{\alpha }}_{i}=\arg\min_{\alpha_{i}}\frac{1}{2}{∥r_{i}-D\alpha_{i}∥}_{2}^{2}+\frac{\lambda }{\mu_{1}}{∥w_{i}\alpha_{i}∥}_{p},$$

where $r_{i}=x_{i}-c_{i}$. Apparently, Equation (21) can be regarded as a sparse coding problem, which can be solved by a variety of non-convex algorithms [50,56,57,58]. However, we can see that how to devise an effective dictionary is quite important in solving $A_{G}$ sub-problem. In general, we can learn an over-complete dictionary with a high computational complexity from natural image dataset [28,30]. However, SC over an over-complete dictionary is potentially unstable and tends to produce visual annoying blocking artifacts in image restoration [33]. In this PSC problem, to achieve a more stable and sparser representation for each patch, we learn the principle component analysis (PCA)-based sub-dictionaries [59] for solving $A_{G}$ sub-problem. Specifically, we first define $R=X_{G}-C$ in Equation (15) as a good approximation of $DA_{G}$. Secondly, we extract image patches from the observation R and use *K*-means algorithm to generate *q* clusters [6]. Finally, we learn *q* PCA sub-dictionaries from each cluster, namely $D=[D_{1},\ldots ,D_{j}],\forall j=1,\ldots ,q$, and then one PCA sub-dictionary is adaptively selected for a given patch.

Now, recalling Equation (21), a generalized soft-thresholding (GST) algorithm [57] is developed to solve it, which is more efficient to implement and converges to a more accurate solution. To be concrete, for fixed D, λ, $w_{i}$ and *p*, the solution of Equation (21) can be computed as

(22) $${\hat{\alpha }}_{i}=\mathrm{GST}\left(D^{-1}r_{i},\frac{\lambda }{\mu_{1}}w_{i},p\right).$$

For more details about the GST algorithm, please refer to [57]. This procedure is applied to all patches for achieving ${\hat{A}}_{G}$, which is the final solution to $A_{G}$ sub-problem in Equation (15). In addition, for the details setting of the weight $w_{i}$, please refer to Section 3.3.

#### 3.2.3. ${\bar{B}}_{G}$ Sub-Problem

Given $X_{G}$, and according to Equation (16), ${\bar{B}}_{G}$ sub-problem can be rewritten as

(23) $${\hat{\bar{B}}}_{G}=\arg\min_{{\bar{B}}_{G}}\frac{1}{2}∥R_{G}-D_{G}{\bar{B}}_{G}{∥}_{F}^{2}+\frac{\rho }{\mu_{2}}{∥K_{G}\circ {\bar{B}}_{G}∥}_{p},$$

where $R_{G}=X_{G}-J$.

Recalling the relationship of ${\bar{B}}_{G}$, $B_{G}$, $B_{G_{i}}$ and $\beta_{i}$, for each patch, we can obtain the other three after solving any one of them. Now, instead of considering each patch as basic unit in the $A_{G}$ sub-problem, we consider each patch-group here. For *i*-th patch-group, we have the following minimization problem,

(24) $${\hat{B}}_{G_{i}}=\arg\min_{B_{G_{i}}}\frac{1}{2}∥R_{G_{i}}-D_{G_{i}}B_{G_{i}}{∥}_{F}^{2}+\frac{\rho }{\mu_{2}}{∥K_{G_{i}}\circ B_{G_{i}}∥}_{p},$$

where $K_{G_{i}}$ is a weight assigned to each patch-group $R_{G_{i}}$. Each weight matrix $K_{G_{i}}$ can enhance the representation ability of each group sparse coefficient $B_{G_{i}}$ [51]. Similar to $A_{G}$ sub-problem, one important issue of solving the sub-problem $B_{G}$ is the selection of the dictionary. In the ${\bar{B}}_{G}$ sub-problem, to better adapt to image local structures, instead of learning an over-complete dictionary for each patch-group as in [18] and inspired by [10,60,61], we learn a singular value decomposition (SVD)-based sub-dictionary for each patch-group. Specifically, we apply the singular value decomposition (SVD) to $R_{G_{i}}$,

(25) $$R_{G_{i}}=U_{G_{i}}G_{G_{i}}V_{G_{i}}^{T}=\sum_{j=1}^{c}g_{i,j}u_{i,j}v_{i,j}^{T},$$

where $G_{G_{i}}=\mathrm{diag}\left(g_{i,1},\ldots ,g_{i,c}\right)$ is a diagonal matrix, c=min(b,m), ∀j=1,…,c, and $u_{i,j},v_{i,j}$ are the columns of $U_{G_{i}}$ and $V_{G_{i}}$, respectively.

Following this, we define each dictionary atom $d_{i,j}$ of the adaptive dictionary $D_{G_{i}}$ for each group $R_{G_{i}}$, i.e.,

(26) $$d_{i,j}=u_{i,j}v_{i,j}^{T},\;\;\;j=1,\ldots ,c,$$

We have therefore learned an adaptive dictionary, i.e.,

(27) $$D_{G_{i}}=\left[d_{i,1},\ldots ,d_{i,c}\right].$$

One can observe that the devised SVD-based dictionary learning method only needs one SVD operation per *patch-group*.

Due to the orthogonality of the dictionary $D_{G_{i}}$, Equation (24) can be rewritten as

(28) $$\begin{array}{cc} {\hat{B}}_{G_{i}} & =\min_{B_{G_{i}}}\left(\frac{1}{2}∥G_{G_{i}}-B_{G_{i}}{∥}_{F}^{2}+\frac{\rho }{\mu_{2}}{∥K_{G_{i}}\circ B_{G_{i}}∥}_{p}\right)\\ \; & =\min_{\beta_{i}}\left(\frac{1}{2}∥g_{i}-\beta_{i}{∥}_{2}^{2}+\frac{\rho }{\mu_{2}}{∥k_{i}\beta_{i}∥}_{p}\right), \end{array}$$

where $R_{G_{i}}=D_{G_{i}}G_{G_{i}}$, and $g_{i},\beta_{i}$ and $k_{i}$ denote the vectorization form of the matrix $G_{G_{i}},B_{G_{i}}$ and $K_{G_{i}}$, respectively.

To achieve the solution of Equation (28) effectively, we invoke the aforementioned GST algorithm [57] to solve it. To be concrete, a closed-form solution of Equation (28) can be achieved as follows:

(29) $${\hat{\beta }}_{i}=\mathrm{GST}\left(g_{i},\frac{\rho }{\mu_{2}}k_{i},p\right).$$

This process is performed across all *n* patch groups to achieve ${\hat{B}}_{G}$, which is the final solution to ${\bar{B}}_{G}$ sub-problem in Equation (16).

### 3.3. Setting the Weight and Regularization Parameter

Inspired by [51], large weights could be used to discourage nonzero entries in the recovered signal, while small weights could be used to encourage nonzero entries. In other words, the weights are inversely proportional to the magnitudes of the sparse coefficients. Therefore, we set the weight $w_{i}$ in $A_{G}$ sub-problem as follows:

(30) $$w_{i}=\frac{1}{|\alpha_{i}|+\varepsilon_{P}},$$

where $\varepsilon_{P}$ is a positive constant.

Similarly, the weight $K_{G_{i}}$ in ${\bar{B}}_{G}$ sub-problem is set as

(31) $$K_{G_{i}}=\frac{1}{|B_{G_{i}}|+\varepsilon_{G}},$$

where $\varepsilon_{G}$ is a positive constant.

Both λ and ρ are regularization parameters. In this paper, to make the proposed image restoration algorithm steadily, we adaptively set the regularization parameters λ and ρ. Specifically, inspired by [62], λ in Equation (21) for $A_{G}$ sub-problem is set to

(32) $$\lambda =\frac{\eta 2\sqrt{2}\sigma_{n}^{2}}{\delta_{i}+\epsilon_{P}},$$

where $\sigma_{n}$ represents the noise variance. $\delta_{i}$ denotes the estimated standard variance of the sparse coefficients of nonlocal similar patches in *j*-th cluster [6] and $\eta ,\epsilon_{P}$ are the positive constants.

Similar to the setting of λ, the parameter ρ in Equation (24) for ${\bar{B}}_{G}$ sub-problem is set as

(33) $$\rho =\frac{\tau 2\sqrt{2}\sigma_{n}^{2}}{\sigma_{i}+\epsilon_{G}},$$

where $\sigma_{i}$ is the estimated standard variance of the group sparse coefficient of each patch-group $R_{G_{i}}$. $\tau ,\epsilon_{P}$ are the positive constants.

### 3.4. Summary of the Proposed Algorithm

Up to now, we have solved the above three sub-problems $X_{G}$, $A_{G}$ and ${\bar{B}}_{G}$ of the proposed SPG-SC model using a non-convex generalized iteration shrinkage algorithm under the framework of ADMM. In practice we can achieve the effective solution for each separated sub-problem, which can guarantee the whole algorithm more efficient and effective. The complete description of the proposed dual-weighted $\ell_{p}$ minimization-based SPG-SC model for image restoration is exhibited in Algorithm 1.
**Algorithm 1** Image Restoration Using SPG-SC Model.**Require:** 
The observed image y and measurement matrix H.   1:Initial ${X_{G}}_{0}=0$, ${A_{G}}_{0}=0$, ${\bar{B}}_{G}=0$, C=0 and J=0.   2:Set parameters *b*, *m*, *L*, $\mu_{1}$, $\mu_{2}$, η, τ, *p*, *t*, $\sigma_{n}$, $\varepsilon_{P}$, $\varepsilon_{G}$, $\epsilon_{P}$ and $\epsilon_{G}$.   3:**for**t=0 Max-Iter **do**   4: Update ${X_{G}}^{\left(t+1\right)}$ by Equation (20);   5: $R^{\left(t+1\right)}={X_{G}}^{\left(t+1\right)}-C^{\left(t\right)}$;   6: Construct dictionary D by $R^{\left(t+1\right)}$ using K-means algorithm and PCA.   7: **for** Each patch $r_{i}$ **do**   8:  Choose the best match PCA dictionary $D_{i}$ for $r_{i}$;   9:  Compute $\alpha_{i}^{\left(t\right)}$ by $D_{i}^{-1}r_{i}$; 10:  Update $w_{i}^{\left(t+1\right)}$ by computing Equation (30); 11:  Update λ by computing Equation (32); 12:  Update $\alpha_{i}^{t+1}$ by computing Equation (22); 13: **end for** 14: $R_{G}^{\left(t+1\right)}=X_{G}^{\left(t+1\right)}-J^{\left(t\right)}$; 15: **for** Each patch-group $R_{G_{i}}$ **do** 16:  Construct dictionary $D_{G_{i}}$ by computing Equation (27); 17:  Compute $B_{G_{i}}^{\left(t\right)}$ by $D_{G_{i}}^{-1}R_{G_{i}}$; 18:  Update $K_{G_{i}}^{\left(t+1\right)}$ by computing Equation (31); 19:  Update ρ by computing Equation (33); 20:  Update $B_{G_{i}}^{\left(t+1\right)}$ by computing Equation (29); 21: **end for** 22: Update ${A_{G}}^{\left(t+1\right)}$ by concatenating all $\alpha_{i}$; 23: Update $D^{\left(t+1\right)}$ by concatenating all $D_{i}$; 24: Update ${{\bar{B}}_{G}}^{\left(t+1\right)}$ by concatenating all $B_{G_{i}}$; 25: Update $D_{G}^{\left(t+1\right)}$ by concatenating all $D_{G_{i}}$; 26: Update $C^{\left(t+1\right)}$ by Equation (17); 27: Update $J^{\left(t+1\right)}$ by Equation (18); 28:**end for** 29:Output: The final restored image $\hat{x}$ by aggregating patches in $X_{G}$.

## 4. Experimental Results

**Figure 1:**
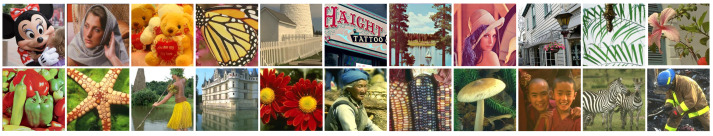
The 22 color images used in our experiments. **Top** row, from left to right: Mickey, Barbara, Bear, Butterfly, Fence, Haight, Lake, Lena, Light, Leaves, Lily. **Bottom** row, from left to right: Pepper, Starfish, Man, Tower, Flowers, Nanna, Corn, Agaric, Monk, Zebra, Fireman.

In this section, extensive experimental results are reported to illustrate the effectiveness of the proposed SPG-SC-based image restoration algorithm. We consider two standard image restoration problems including image inpainting and image deblurring. The experimental test images are shown as in Figure 1. Both peak signal to noise ratio (PSNR) and structural similarity (SSIM) [63] are used to evaluate different image restoration algorithms objectively. The following objective function is chosen as the stopping criterion for the proposed SPG-SC-based image restoration algorithm, i.e.,

(34) $$\frac{∥{\hat{x}}^{t}-{\hat{x}}^{t-1}{∥}_{2}^{2}}{∥{\hat{x}}^{t-1}{∥}_{2}^{2}}<\xi ,$$

where ξ is a small tolerance. The source code of our SPG-SC-based image restoration algorithm is available at: https://drive.google.com/open?id=1-nD7Mkb6Kn1TWzzxk5Pg886loIX_yb8P.

### 4.1. Parameter Setting

Before giving the experimental results, we first briefly introduce the parameter setting of the proposed image restoration algorithms. Specifically, we consider two interesting scenarios in image inpainting, i.e., random pixel corruption and text inlayed. The parameters of the proposed SPG-SC model for image inpainting are set as follows. The size of each patch $\sqrt{b}\times \sqrt{b}$ is set to 8×8. The searching window of similar patches L×L is set to 25×25, $\sigma_{n}=\sqrt{2}$ and the number of similar patches *m* is set to 60. Four small constants ($\varepsilon_{P}$, $\varepsilon_{G}$, $\epsilon_{P}$, $\epsilon_{G}$) are set to (0.1, 0.1, $e^{-14}$, 0.4) to avoid dividing by zero. The parameters ($\mu_{1}$, $\mu_{2}$, η, τ, *p*, ξ) are set to (0.00009, 0.0007, 0.8, 0.6, 0.7, 0.0030), (0.0001, 0.0007, 0.4, 1.1, 0.6, 0.0032), (0.0001, 0.0009, 1, 1, 0.55, 0.0024), (0.0002, 0.01, 0.9, 0.3, 1, 0.0004), (0.0001, 0.04, 0.7, 0.9, 1, 0.0001) and (0.0001, 0.0007, 0.4, 1.1, 0.6, 0.0015) for 90%, 80%, 70%, 60%, 50% pixels missing and text inlayed, respectively.

In image deblurring, two types of blur kernels are considered in this paper, i.e., 9×9 uniform kernel and a Gaussian kernel with standard deviation 1.6. Each blurred image is generated using a blur kernel to the original image first, and then followed by adding an additive white Gaussian noise with standard variance $\sigma_{n}=\sqrt{2}$. The parameter setting of our proposed SPG-SC model for image deblurring is as follows. The size of each patch $\sqrt{b}\times \sqrt{b}$ is set to 8×8 and the searching window L×L is set to 20×20. The searching similar patches *m* is set to 60. Four small constants ($\varepsilon_{P}$, $\varepsilon_{G}$, $\epsilon_{P}$, $\epsilon_{G}$) are as the same setting as the image inpainting task. The parameters ($\mu_{1}$, $\mu_{2}$, η, τ, *p*, ξ) are set to (0.0003, 0.03, 0.2, 1.2, 0.75, 0.00018) and (0.0001, 0.02, 0.1, 0.8, 0.9, 0.00012) for uniform kernel and Gaussian kernel, respectively. Moreover, we will provide a detailed discussion on how to choose the best power *p* in Section 4.5.

### 4.2. Image Inpainting

In this subsection, we apply the proposed SPG-SC algorithm to image inpainting task. We compare it with nine advanced methods, including BPFA [64], IPPO [65], ISD-SB [66], JSM [7], Aloha [60], NGS [67], BKSVD [68], WNNM [40] and TSLRA [69]. It is worth nothing that nonlocal redundancies are used for IPPO, JSM, Aloha, NGS, WNNM and TSLRA methods. BKSVD is a typical PSC method, while JSM and NGS are based on the GSC models. WNNM (In this paper, we employ the WNNM model along with ADMM algorithm to image inpainting and image deblurring tasks.) exploits low-rank prior via nuclear norm and achieves a state-of-the-art denoising result. TSLRA is also a low-rank method that delivers the state-of-the-art image inpainting performance.

The PSNR and SSIM comparison results for a collection of 13 color images in the case of 80%, 70%, 60%, 50% pixels missing and text inlayed are shown in Table 1 and Table 2, respectively, with the best results highlighted in bold. As can be seen from Table 1, the proposed SPG-SC achieves better results than the other image inpainting methods. On average, our proposed SPG-SC enjoys a PSNR performance gain over BPFA by 2.79 dB, over IPPO by 1.26 dB, over ISD-SB by 6.22 dB, over JSM by 1.42 dB, over Aloha by 1.86 dB, over NGS by 3.56 dB, over BKSVD by 3.52 dB, over WNNM by 0.45dB and over TSLRA by 1.82 dB. In particular, one can observe that under the conditions of 80%, 70%, 60% and 50% pixels missing, the proposed SPG-SC consistently outperforms the other competing methods for all test images in terms of PSNR. Regarding SSIM, the proposed SPG-SC also performs better performance than all competing methods in most cases. To be concrete, in terms of SSIM, the proposed SPG-SC achieves 0.0398, 0.0122, 0.1135, 0.0169, 0.0216, 0.0457, 0.0600, 0.0060 and 0.0193 gain on average over BPFA, IPPO, ISD-SB, JSM, Aloha, NGS, BKSVD, WNNM and TSLRA for all cases, respectively.

**Figure 2:**
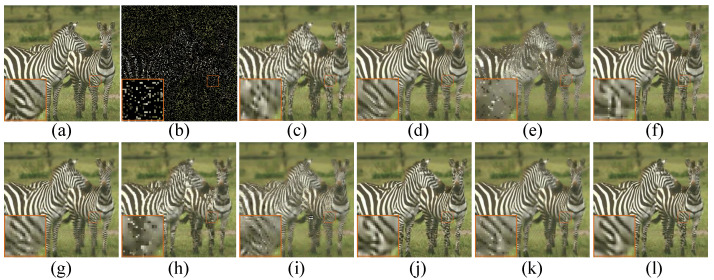
Visual comparison of *Zebra* by image inpainting with 80% missing pixels. (**a**) Original image; (**b**) Degraded image with 80% pixels missing; (**c**) BPFA [64] (PSNR = 20.90 dB, SSIM = 0.7160); (**d**) IPPO [65] (PSNR = 22.71 dB, SSIM = 0.7744); (**e**) ISD-SB [66] (PSNR = 18.41 dB, SSIM = 0.5899); (**f**) JSM [7] (PSNR = 21.88 dB, SSIM = 0.7556); (**g**) Aloha [60] (PSNR = 22.72 dB, SSIM = 0.7720); (**h**) NGS [67] (PSNR = 20.49 dB, SSIM = 0.7132); (**i**) BKSVD [68] (PSNR = 19.37 dB, SSIM = 0.6912); (**j**) WNNM [40] (PSNR = 22.67 dB, SSIM = 0.7958); (**k**) TSLRA [69] (PSNR = 22.37 dB, SSIM = 0.7572); (**l**) SPG-SC (PSNR = **23.06 dB**, SSIM = **0.7966**).

**Figure 3:**
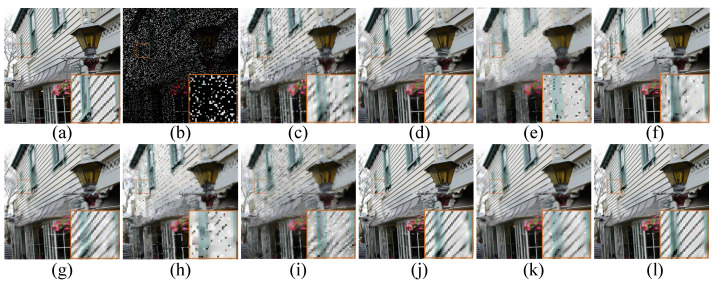
Visual comparison of *Light* by image inpainting with 80% missing pixels. (**a**) Original image; (**b**) Degraded image with 80% pixels missing; (**c**) BPFA [64] (PSNR = 19.26 dB, SSIM = 0.6285); (**d**) IPPO [65] (PSNR = 21.49 dB, SSIM = 0.7827); (**e**) ISD-SB [66] (PSNR = 17.48 dB, SSIM = 0.4902); (**f**) JSM [7] (PSNR = 20.23 dB, SSIM = 0.7254); (**g**) Aloha [60] (PSNR = 21.50 dB, SSIM = 0.7734); (**h**) NGS [67] (PSNR = 18.52 dB, SSIM = 0.6041); (**i**) BKSVD [68] (PSNR = 18.77 dB, SSIM = 0.5792); (**j**) WNNM [40] (PSNR = 22.09 dB, SSIM = 0.8236); (**k**) TSLRA [69] (PSNR = 21.73 dB, SSIM = 0.7780); (**l**) SPG-SC (PSNR = **22.43 dB**, SSIM = **0.8318**).

**Figure 4:**
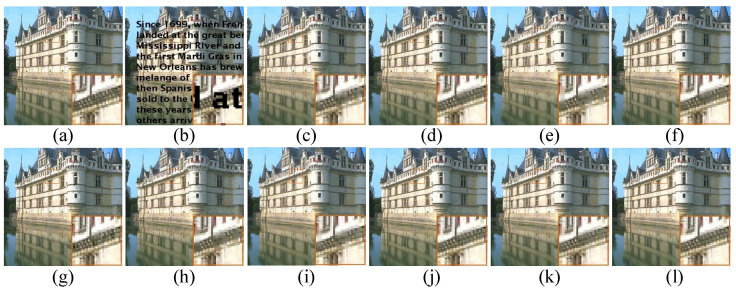
Visual comparison of *Tower* by image inpainting with text inlayed. (**a**) Original image; (**b**) Degraded image with 80% pixels missing; (**c**) BPFA [64] (PSNR = 30.94 dB, SSIM = 0.9530); (**d**) IPPO [65] (PSNR = 31.91 dB, SSIM = 0.9685); (**e**) ISD-SB [66] (PSNR = 28.81 dB, SSIM = 0.9345); (**f**) JSM [7] (PSNR = 32.48 dB, SSIM = 0.9696); (**g**) Aloha [60] (PSNR = 30.34 dB, SSIM = 0.9550); (**h**) NGS [67] (PSNR = 30.21 dB, SSIM = 0.9465); (**i**) BKSVD [68] (PSNR = 30.35 dB, SSIM = 0.9413); (**j**) WNNM [40] (PSNR = 32.70 dB, SSIM = 0.9727); (**k**) TSLRA [69] (PSNR = 31.43 dB, SSIM = 0.9633); (**l**) SPG-SC (PSNR = **33.23 dB**, SSIM = **0.9728**).

Apart from the objective measurements mentioned above, human subject perceptivity is judging of image quality ultimately, which is also critical to evaluate an image restoration algorithm. We show the visual comparisons of the images *Zebra* and *Light* with 80% pixels missing in Figure 2 and Figure 3, respectively. Meanwhile, the visual comparison of image Tower with text inlayed is shown in Figure 4. On the whole, we can observe that BPFA, ISD-SB, JSM, NGS and BKSVD methods are all prone to produce some visual annoying blocking artifacts, while IPPO, Aloha, WNNM and TSLRA methods often over-smooth the images and lose some details. By contrast, our proposed SPG-SC approach can preserve fine details and suppress undesirable visual artifacts more effective than all competing methods.

### 4.3. Image Deblurring

In this subsection, we describe the experimental results of the proposed SPG-SC-based image deblurring. We compare it with leading non-blind deblurring methods, including BM3D [70], L0-ABS [71], ASDS [59], EPLL [72], NCSR [6], JSM [7], L2-r-L0 [73], WNNM [40] and NLNCDR [74]. Please note that BM3D, ASDS, EPLL, NCSR, JSM, WNNM and NLNCDR are using the image NSS priors. BM3D is a well-known one that uses the GSC model for image restoration and delivers state-of-the-art denoising results. NCSR exploits a sparsity residual model under the GSC framework, which is one of the state-of-the-art image deblurring algorithms. ASDS and JSM jointly consider local sparsity and nonlocal sparsity constraints.

As can be seen from Table 3, we report the PSNR and SSIM results for a collection of 14 color images for these approaches. It can be observed that our proposed SPG-SC achieves better PSNR and SSIM results than the other competing methods in most cases. Specifically, on average, our proposed SPG-SC achieves {0.69 dB, 1.08 dB, 0.53 dB, 3.61 dB, 0.16 dB, 1.65 dB, 0.66 dB, 0.09 dB and 0.99 dB} gains in PSNR and {0.0267, 0.0317, 0.0307, 0.0456, 0.0107, 0.0989, 0.0198, 0.0047 and 0.0482} gains in SSIM over BM3D, L0-ABS, ASDS, EPLL, NCSR, JSM, L2-r-L0, WNNM and NLNCDR for all cases, respectively.

**Figure 5:**
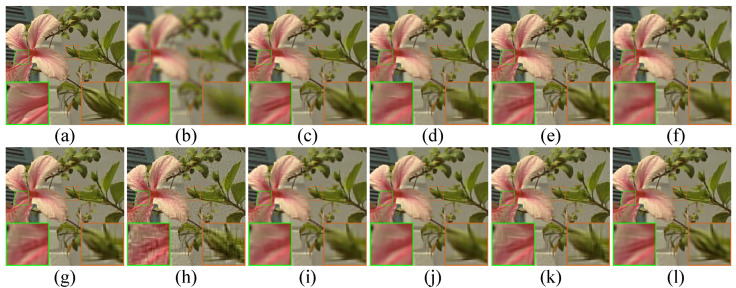
Visual comparison of *Lily* by image deblurring with uniform kernel. (**a**) Original image; (**b**) noisy and blurred image (9×9 uniform kernel, $\sigma_{n}\;=\;\sqrt{2}$); (**c**) BM3D [70] (PSNR = 28.58 dB, SSIM = 0.8119); (**d**) L0-ABS [71] (PSNR = 28.05 dB, SSIM = 0.8004); (**e**) ASDS [59] (PSNR = 29.21 dB, SSIM = 0.8290); (**f**) EPLL [72] (PSNR = 27.04 dB, SSIM = 0.7981); (**g**) NCSR [6] (PSNR = 29.39 dB, SSIM = 0.8393); (**h**) JSM [7] (PSNR = 26.97 dB, SSIM = 0.6924); (**i**) L2-r-L0 [73] (PSNR = 28.47 dB, SSIM = 0.8155); (**j**) WNNM [40] (PSNR = 29.25 dB, SSIM = 0.8406); (**k**) NLNCDR [74] (PSNR = 28.69 dB, SSIM = 0.8039); (**l**) SPG-SC (PSNR = **29.40 dB**, SSIM = **0.8476**).

**Figure 6:**
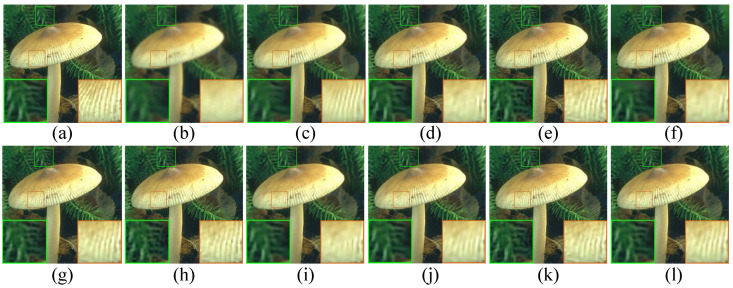
Visual comparison of *Agaric* by image deblurring with Gaussian kernel. (**a**) Original image; (**b**) noisy and blurred image (fspecial(‘gaussian’, 25, 1.6), $\sigma_{n}\;=\;\sqrt{2}$); (**c**) BM3D [70] (PSNR = 30.34 dB, SSIM = 0.8368); (**d**) L0-ABS [71] (PSNR = 30.07 dB, SSIM = 0.8392); (**e**) ASDS [59] (PSNR = 30.06 dB, SSIM = 0.8113); (**f**) EPLL [72] (PSNR = 27.69 dB, SSIM = 0.8118); (**g**) NCSR [6] (PSNR = 30.56 dB, SSIM = 0.8466); (**h**) JSM [7] (PSNR = 29.96 dB, SSIM = 0.8087); (**i**) L2-r-L0 [73] (PSNR = 30.30 dB, SSIM = 0.8458); (**j**) WNNM [40] (PSNR = 30.68 dB, SSIM = 0.8570); (**k**) NLNCDR [74] (PSNR = 29.92 dB, SSIM = 0.8081); (**l**) SPG-SC (PSNR = **30.84 dB**, SSIM = **0.8603**).

We also show the visual comparison results of image deblurring from Figure 5 to Figure 6. One can clearly see that ASDS, NCSR, JSM and NLNCDR methods still suffer from some undesirable visual blocking artifacts, such as ring artifacts. At the same time, BM3D, L0-ABS, EPLL, L2-r-L0 and WNNM methods are apt to generate the over-smooth effect, such as some details are lost in the recovered images. Compared to the competing methods, the proposed SPG-SC not only produces visually pleasant results, but also preserves image details and textures with a higher accuracy.

In summary, our proposed SPG-SC approach seems to keep a good balance between artifact removal and detail preservations, which is attributed to the following aspects: (1) the local sparsity and nonlocal sparse representation are simultaneously considered in our proposed SPG-SC method; (2) a dual-weighted $\ell_{p}$ minimization further enhances the sparse representation capability of our proposed SPG-SC model; (3) we have developed two sub-dictionaries to better adapt to image local structures.

### 4.4. Algorithm Convergence

**Figure 7:**
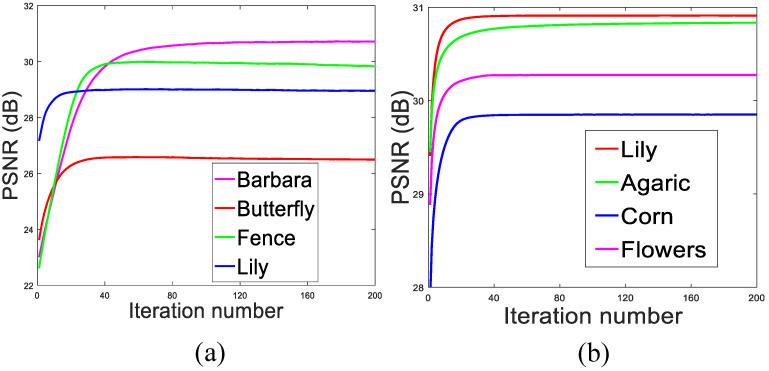
Convergence analysis of the proposed algorithm. (**a**) PSNR results versus iteration number for image inpainting with 80% pixels missing. (**b**) PSNR results versus iteration number for image deblurring with Gaussian kernel.

Since the objective function of Equation (13), comprising two $\ell_{p}$ norms, is non-convex, it is quite difficult to provide its theoretical proof for global convergence. In this subsection, we give empirical evidence to illustrate the convergence of our proposed SPG-SC algorithm. Concretely, the convergence of the proposed SPG-SC algorithm is shown in Figure 7, where Figure 7a plots the evolutions of PSNR values versus the iteration numbers for image inpainting with 80% pixels missing (including image *Barbara*, *Butterfly*, *Fence* and *Lily*), and Figure 7b plots the evolutions of PSNR values versus the iteration numbers for image deblurring with Gaussian kernel (including image *Lily*, *Agaric*, *Corn* and *Flowers*). We can clearly see that with the growth of iteration numbers, the PSNR curves of the recovered images monotonically increase and ultimately become flat and stable. Thus, we conclude that the proposed SPG-SC algorithm exhibits a good convergence performance.

### 4.5. Suitable Setting of the Power *p*

**Figure 8:**
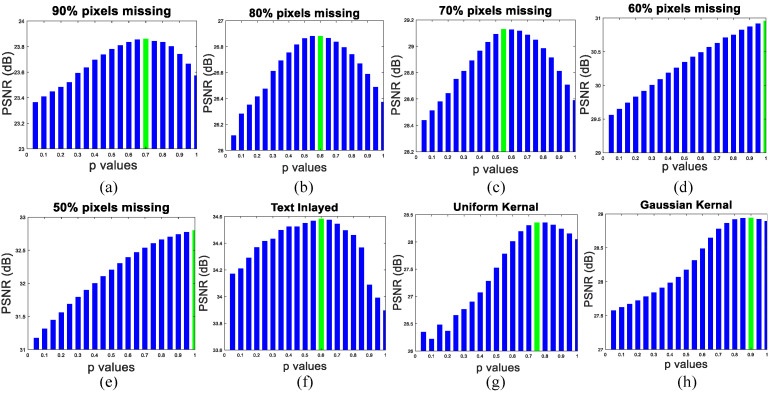
Testing the different power *p* value for the influence of image restoration tasks. (**a**–**f**) PSNR values versus *p* for image inpainting; (**g**,**h**) PSNR values versus *p* for image deblurring.

In this subsection, we present how to obtain the best performance of our proposed SPG-SC-based image restoration algorithm through selecting the suitable power *p* value. To show the influence of the power *p* value introduced by the proposed dual-weighted $\ell_{p}$ minimization, we randomly choose 20 images (size: 256×256) from the Berkeley Segmentation Dataset 200 (BSD200) [75] as test images and conduct our proposed algorithm with different *p* values to image restoration tasks including image inpainting and image deblurring. The average PSNR results under different power *p* values from 0.05 to 1 with interval 0.05 are shown in Figure 8. It can be found from Figure 8a to Figure 8f that the best PSNR results of image inpainting for our proposed algorithm is achieved by selecting *p* = 0.70, 0.60, 0.55, 1, 1 and 0.60 when 90%, 80%, 70%, 60%, 50% pixels missing and text inlayed, respectively. From Figure 8g to Figure 8h, we can observe that the best reconstructed performance of image deblurring is obtained by choosing *p* = 0.75 and 0.90 for uniform kernel and Gaussian kernel, respectively. Therefore, in image inpainting, we choose *p* = 0.70, 0.60, 0.55, 1, 1 and 0.60 for 90%, 80%, 70%, 60%, 50% pixels missing and text inlayed, respectively. In image deblurring, we choose *p* = 0.75 and 0.90 for uniform kernel and Gaussian kernel, respectively.

## 5. Conclusions

This paper proposed a novel method for image restoration via simultaneous patch-group sparse coding (SPG-SC) with dual-weighted $\ell_{p}$ minimization. Compared with the existing sparse coding-based methods, the proposed SPG-SC considered the local sparsity and nonlocal sparse representation simultaneously. We have proposed a dual-weighted $\ell_{p}$ minimization-based non-convex regularization to improve the sparse representation capability of the proposed SPG-SC model. Two sub-dictionaries have been used to better adapt to image local structures, rather than learning an over-complete dictionary with a high computational complexity from natural image dataset. To make the optimization tractable, we have developed a non-convex generalized iteration shrinkage algorithm based on the alternating direction method of multipliers (ADMM) framework to solve the proposed SPG-SC model. Experimental results on two image restoration applications including image inpainting and image deblurring, have demonstrated that the proposed SPG-SC achieves better results than many state-of-the-art algorithms and exhibits a good convergence property.

## Figures and Tables

**Table 1:** PSNR (dB) comparison of BPFA [64], IPPO [65], ISD-SB [66], JSM [7], Aloha [60], NGS [67], BKSVD [68], WNNM [40], TSLRA [69] and SPG-SC for image inpainting.

	Pixels Missing = 80%													
**Images**	**Mickey**	**Barbara**	**Butterfly**	**Fence**	**Haight**	**Leaves**	**Lena**	**Light**	**Lily**	**Pepper**	**Starfish**	**Tower**	**Zebra**	**Average**
BPFA [64]	24.53	25.11	24.04	26.24	19.42	23.78	29.50	19.26	27.30	29.58	26.79	23.94	20.90	24.65
IPPO [65]	26.33	28.32	25.13	27.98	20.90	25.56	30.64	21.49	28.33	30.48	26.30	24.50	22.71	26.05
ISD-SB [66]	22.25	22.35	18.57	21.39	17.00	18.70	26.01	17.48	24.53	25.19	23.00	21.47	18.41	21.26
JSM [7]	26.09	26.95	25.57	28.59	21.37	26.18	30.46	20.23	27.99	30.48	27.07	24.59	21.88	25.96
Aloha [60]	25.33	29.59	24.88	28.88	20.62	25.90	30.89	21.50	27.70	29.95	26.33	23.88	22.72	26.01
NGS [67]	24.50	23.88	23.85	25.26	18.76	23.87	28.87	18.52	27.08	29.35	26.17	23.47	20.49	24.16
BKSVD [68]	23.72	25.21	22.00	24.20	18.83	22.05	28.16	18.77	26.49	27.75	25.36	22.93	19.37	23.45
WNNM [40]	26.66	30.49	26.46	29.74	21.43	27.10	30.99	22.09	28.94	30.74	27.66	24.60	22.67	26.89
TSLRA [69]	25.71	28.22	25.32	28.83	20.85	25.47	30.58	21.73	28.17	29.31	26.84	24.26	22.37	25.97
SPG-SC	**26.75**	**30.69**	**26.47**	**29.80**	**21.83**	**27.16**	**31.41**	**22.43**	**28.94**	**31.55**	**28.03**	**24.64**	**23.06**	**27.14**
	Pixels Missing = 70%													
**Images**	**Mickey**	**Barbara**	**Butterfly**	**Fence**	**Haight**	**Leaves**	**Lena**	**Light**	**Lily**	**Pepper**	**Starfish**	**Tower**	**Zebra**	**Average**
BPFA [64]	26.16	28.32	26.68	28.87	21.46	26.98	31.62	21.58	29.30	31.74	28.93	25.66	22.78	26.93
IPPO [65]	28.59	30.89	27.68	30.08	23.02	28.58	32.97	23.47	30.28	33.05	28.91	26.11	24.76	28.34
ISD-SB [66]	24.40	23.56	22.65	23.16	18.89	21.85	28.16	18.70	26.46	28.37	25.09	23.18	20.17	23.43
JSM [7]	28.25	30.48	27.97	30.46	23.01	29.28	32.69	23.12	29.83	33.47	29.36	26.64	23.95	28.35
Aloha [60]	27.11	32.40	27.29	30.57	22.12	29.04	32.80	23.17	29.58	32.76	28.22	25.77	24.55	28.11
NGS [67]	26.68	26.11	26.36	27.32	21.03	26.44	30.77	20.78	28.83	31.59	28.35	25.22	22.71	26.32
BKSVD [68]	26.17	27.58	25.00	28.35	21.12	25.29	30.96	20.85	28.65	30.96	27.79	25.07	23.06	26.22
WNNM [40]	29.16	33.05	29.19	31.55	23.56	30.55	33.32	24.00	30.79	33.49	30.07	26.61	24.75	29.24
TSLRA [69]	27.64	30.79	27.76	30.75	22.61	28.03	32.64	23.43	29.92	32.72	28.78	26.05	24.25	28.11
SPG-SC	**29.29**	**34.20**	**29.22**	**31.80**	**23.96**	**30.64**	**33.68**	**24.30**	**31.26**	**34.79**	**30.57**	**26.81**	**25.22**	**29.67**
	Pixels Missing = 60%													
**Images**	**Mickey**	**Barbara**	**Butterfly**	**Fence**	**Haight**	**Leaves**	**Lena**	**Light**	**Lily**	**Pepper**	**Starfish**	**Tower**	**Zebra**	**Average**
BPFA [64]	27.83	31.06	28.88	30.79	23.33	29.83	33.54	23.62	31.35	34.20	30.98	27.28	24.53	29.02
IPPO [65]	30.76	33.55	29.85	32.14	25.34	30.88	34.89	25.13	32.17	35.16	31.09	27.81	26.79	30.43
ISD-SB [66]	26.59	24.86	25.07	25.30	21.02	24.55	30.52	19.81	28.23	30.68	27.36	24.95	22.34	25.48
JSM [7]	29.85	33.21	29.83	32.23	24.70	31.47	34.56	24.83	31.59	35.47	31.40	28.09	25.90	30.24
Aloha [60]	28.59	35.13	29.16	32.33	23.58	31.41	34.72	24.47	31.47	35.00	30.19	27.16	26.24	29.96
NGS [67]	28.09	28.24	28.37	30.11	22.81	28.87	32.81	22.78	30.53	33.59	30.26	27.04	24.39	28.30
BKSVD [68]	28.53	29.86	27.70	30.72	23.39	28.61	33.48	23.00	31.00	33.44	29.99	26.68	25.27	28.59
WNNM [40]	31.23	35.61	31.27	33.18	25.87	32.89	35.06	25.43	32.80	35.49	32.28	28.10	27.07	31.25
TSLRA [69]	29.28	33.37	29.42	32.32	24.21	30.19	34.26	24.80	31.55	34.96	30.69	27.60	25.92	29.89
SPG-SC	**31.44**	**36.82**	**31.60**	**33.66**	**26.34**	**33.56**	**36.01**	**25.68**	**33.34**	**36.87**	**33.05**	**28.55**	**27.33**	**31.87**
	Pixels Missing = 50%													
**Images**	**Mickey**	**Barbara**	**Butterfly**	**Fence**	**Haight**	**Leaves**	**Lena**	**Light**	**Lily**	**Pepper**	**Starfish**	**Tower**	**Zebra**	**Average**
BPFA [64]	29.43	34.01	30.98	32.82	25.40	32.79	35.61	25.73	33.41	36.44	33.13	28.83	26.37	31.15
IPPO [65]	32.74	35.91	31.69	33.95	27.53	33.32	36.50	26.70	34.04	36.91	33.10	29.57	28.42	32.34
ISD-SB [66]	27.96	26.57	27.76	27.60	22.92	26.97	32.04	21.17	30.05	32.43	29.16	26.75	23.91	27.33
JSM [7]	31.96	35.87	31.47	33.75	26.67	33.78	36.39	26.48	33.46	37.35	33.24	29.48	27.77	32.13
Aloha [60]	30.33	37.46	30.78	33.79	25.16	34.01	36.41	25.84	33.33	36.88	31.85	28.71	27.67	31.71
NGS [67]	29.75	30.93	30.28	32.00	24.50	31.23	34.56	24.62	32.31	35.59	32.10	28.53	26.03	30.19
BKSVD [68]	29.95	33.58	29.64	32.44	25.23	31.25	35.44	24.68	32.93	35.87	31.99	28.27	26.97	30.63
WNNM [40]	33.67	37.47	33.00	34.53	28.36	35.41	36.80	27.28	34.74	37.26	34.27	29.83	29.07	33.21
TSLRA [69]	31.00	35.74	31.01	33.89	26.02	32.56	35.52	26.27	33.20	36.61	32.44	29.14	27.67	31.62
SPG-SC	**34.00**	**39.11**	**33.25**	**35.22**	**28.90**	**36.26**	**37.81**	**27.37**	**35.42**	**38.60**	**34.98**	**30.20**	**29.33**	**33.88**
	Text Inlayed													
**Images**	**Mickey**	**Barbara**	**Butterfly**	**Fence**	**Haight**	**Leaves**	**Lena**	**Light**	**Lily**	**Pepper**	**Starfish**	**Tower**	**Zebra**	**Average**
BPFA [64]	31.70	34.27	31.71	32.23	26.64	31.78	35.27	28.63	35.18	37.50	33.88	30.94	27.04	32.06
IPPO [65]	34.04	37.65	33.98	35.10	29.10	35.26	37.29	29.92	36.67	39.42	35.35	31.91	29.99	34.28
ISD-SB [66]	29.96	30.43	28.09	27.62	24.61	27.64	33.13	24.94	32.72	34.70	31.38	28.81	24.96	29.15
JSM [7]	32.99	37.79	33.19	35.41	28.69	35.40	36.98	29.65	35.67	39.27	35.17	32.48	29.11	33.98
Aloha [60]	30.49	39.16	31.58	34.94	26.21	34.74	36.03	28.38	34.47	37.40	32.06	30.34	28.87	32.67
NGS [67]	31.10	33.57	31.78	28.73	26.16	30.05	34.71	27.26	34.00	35.61	33.02	30.21	26.24	30.96
BKSVD [68]	31.43	35.16	29.09	31.69	26.59	29.74	34.66	27.77	34.01	34.90	32.83	30.35	27.91	31.24
WNNM [40]	34.51	39.58	34.50	36.25	29.93	**36.34**	37.31	**30.31**	**36.68**	39.73	36.18	32.70	29.86	34.91
TSLRA [69]	32.43	37.78	32.64	35.23	28.21	33.66	32.76	29.41	35.42	37.02	34.50	31.43	28.95	33.03
SPG-SC	**34.87**	**39.94**	**34.54**	**37.07**	**30.43**	36.24	**37.73**	30.26	36.56	**39.84**	**36.27**	**33.23**	**30.46**	**35.19**

**Table 2:** SSIM comparison of BPFA [64], IPPO [65], ISD-SB [66], JSM [7], Aloha [60], NGS [67], BKSVD [68], WNNM [40], TSLRA [69] and SPG-SC for image inpainting.

	Pixels Missing = 80%													
**Images**	**Mickey**	**Barbara**	**Butterfly**	**Fence**	**Haight**	**Leaves**	**Lena**	**Light**	**Lily**	**Pepper**	**Starfish**	**Tower**	**Zebra**	**Average**
BPFA [64]	0.8117	0.8042	0.8517	0.7960	0.7307	0.8557	0.8899	0.6285	0.8234	0.9127	0.8379	0.7790	0.7160	0.8029
IPPO [65]	0.8678	0.8834	0.8995	0.8614	0.8251	0.9119	0.9085	0.7827	0.8587	0.9238	0.8243	0.8217	0.7744	0.8572
ISD-SB [66]	0.7506	0.6442	0.7363	0.5994	0.6403	0.6941	0.8071	0.4902	0.7059	0.8335	0.7035	0.6595	0.5899	0.6811
JSM [7]	0.8598	0.8354	0.9026	0.8530	0.8320	0.9213	0.8988	0.7254	0.8418	0.9224	0.8383	0.8257	0.7556	0.8471
Aloha [60]	0.8300	0.9118	0.8805	0.8699	0.7955	0.9085	0.9095	0.7734	0.8402	0.9177	0.8217	0.8090	0.7720	0.8492
NGS [67]	0.8230	0.7594	0.8635	0.7898	0.7351	0.8687	0.8767	0.6041	0.8174	0.9117	0.8272	0.7783	0.7132	0.7976
BKSVD [68]	0.7713	0.7912	0.7817	0.7833	0.6951	0.7782	0.8500	0.5792	0.7804	0.8759	0.7741	0.7344	0.6912	0.7605
WNNM [40]	0.8738	0.9148	0.9184	0.8717	0.8526	0.9319	0.8968	0.8236	0.8615	0.9146	0.8435	**0.8426**	0.7958	0.8724
TSLRA [69]	0.8536	0.8786	0.8928	0.8679	0.8119	0.9029	0.9001	0.7780	0.8460	0.9166	0.8311	0.8078	0.7572	0.8496
SPG-SC	**0.8770**	**0.9162**	**0.9241**	**0.8780**	**0.8555**	**0.9375**	**0.9171**	**0.8318**	**0.8719**	**0.9353**	**0.8660**	0.8365	**0.7966**	**0.8803**
	Pixels Missing = 70%													
**Images**	**Mickey**	**Barbara**	**Butterfly**	**Fence**	**Haight**	**Leaves**	**Lena**	**Light**	**Lily**	**Pepper**	**Starfish**	**Tower**	**Zebra**	**Average**
BPFA [64]	0.8661	0.8919	0.9124	0.8726	0.8269	0.9276	0.9269	0.7864	0.8856	0.9435	0.8942	0.8519	0.8042	0.8762
IPPO [65]	0.9151	0.9334	0.9356	0.9042	0.8878	0.9538	0.9422	0.8612	0.9088	0.9518	0.8923	0.8771	0.8498	0.9087
ISD-SB [66]	0.8251	0.7259	0.8541	0.7131	0.7430	0.8222	0.8647	0.6222	0.7955	0.8971	0.7957	0.7508	0.7047	0.7780
JSM [7]	0.9064	0.9228	0.9377	0.8996	0.8831	0.9581	0.9354	0.8528	0.8935	0.9534	0.8954	0.8860	0.8344	0.9045
Aloha [60]	0.8797	0.9505	0.9205	0.9105	0.8557	0.9549	0.9420	0.8496	0.8934	0.9493	0.8793	0.8738	0.8432	0.9002
NGS [67]	0.8791	0.8556	0.9145	0.8607	0.8303	0.9233	0.9145	0.7538	0.8728	0.9414	0.8856	0.8478	0.8063	0.8681
BKSVD [68]	0.8509	0.8775	0.8753	0.8615	0.8013	0.8896	0.9094	0.7331	0.8579	0.9264	0.8552	0.8247	0.7941	0.8505
WNNM [40]	0.9195	0.9449	0.9473	0.9098	0.9037	0.9641	0.9358	0.8860	0.9052	0.9454	0.8997	0.8962	0.8584	0.9166
TSLRA [69]	0.8993	0.9298	0.9347	0.9071	0.8717	0.9452	0.9367	0.8502	0.8949	0.9499	0.8862	0.8705	0.8321	0.9006
SPG-SC	**0.9227**	**0.9582**	**0.9520**	**0.9201**	**0.9065**	**0.9696**	**0.9489**	**0.8902**	**0.9208**	**0.9618**	**0.9167**	**0.8965**	**0.8665**	**0.9254**
	Pixels Missing = 60%													
**Images**	**Mickey**	**Barbara**	**Butterfly**	**Fence**	**Haight**	**Leaves**	**Lena**	**Light**	**Lily**	**Pepper**	**Starfish**	**Tower**	**Zebra**	**Average**
BPFA [64]	0.9033	0.9394	0.9436	0.9125	0.8844	0.9615	0.9498	0.8662	0.9260	0.9620	0.9280	0.8971	0.8647	0.9184
IPPO [65]	0.9425	0.9598	0.9566	0.9346	0.9287	0.9726	0.9601	0.9057	0.9394	0.9672	0.9290	0.9146	0.9001	0.9393
ISD-SB [66]	0.8749	0.7969	0.9017	0.7994	0.8260	0.8953	0.9049	0.7097	0.8552	0.9291	0.8587	0.8225	0.7994	0.8441
JSM [7]	0.9327	0.9554	0.9570	0.9296	0.9195	0.9751	0.9557	0.9010	0.9286	0.9682	0.9293	0.9182	0.8856	0.9351
Aloha [60]	0.9127	0.9697	0.9428	0.9385	0.8968	0.9736	0.9594	0.8910	0.9288	0.9657	0.9171	0.9072	0.8899	0.9302
NGS [67]	0.9119	0.9099	0.9451	0.9086	0.8842	0.9556	0.9443	0.8452	0.9115	0.9602	0.9222	0.8982	0.8630	0.9123
BKSVD [68]	0.9050	0.9324	0.9266	0.9075	0.8780	0.9480	0.9409	0.8414	0.9121	0.9515	0.9059	0.8790	0.8626	0.9070
WNNM [40]	0.9450	0.9651	0.9630	0.9376	0.9381	0.9780	0.9525	0.9175	0.9379	0.9604	0.9315	0.9241	0.9060	0.9428
TSLRA [69]	0.9263	0.9577	0.9531	0.9343	0.9104	0.9666	0.9555	0.8934	0.9282	0.9654	0.9231	0.9086	0.8830	0.9312
SPG-SC	**0.9485**	**0.9750**	**0.9677**	**0.9461**	**0.9401**	**0.9840**	**0.9667**	**0.9209**	**0.9500**	**0.9739**	**0.9453**	**0.9282**	**0.9106**	**0.9505**
	Pixels Missing = 50%													
**Images**	**Mickey**	**Barbara**	**Butterfly**	**Fence**	**Haight**	**Leaves**	**Lena**	**Light**	**Lily**	**Pepper**	**Starfish**	**Tower**	**Zebra**	**Average**
BPFA [64]	0.9312	0.9633	0.9617	0.9390	0.9226	0.9795	0.9658	0.9169	0.9516	0.9723	0.9510	0.9260	0.9076	0.9453
IPPO [65]	0.9606	0.9749	0.9697	0.9550	0.9540	0.9832	0.9723	0.9350	0.9599	0.9769	0.9531	0.9413	0.9301	0.9589
ISD-SB [66]	0.9077	0.8562	0.9364	0.8658	0.8792	0.9355	0.9298	0.7968	0.8989	0.9484	0.9002	0.8756	0.8558	0.8913
JSM [7]	0.9537	0.9741	0.9695	0.9502	0.9459	0.9846	0.9707	0.9322	0.9528	0.9775	0.9518	0.9411	0.9218	0.9558
Aloha [60]	0.9371	0.9815	0.9580	0.9555	0.9244	0.9850	0.9719	0.9212	0.9525	0.9764	0.9418	0.9323	0.9197	0.9505
NGS [67]	0.9386	0.9469	0.9630	0.9376	0.9190	0.9734	0.9625	0.8983	0.9408	0.9735	0.9464	0.9296	0.9035	0.9410
BKSVD [68]	0.9302	0.9561	0.9487	0.9317	0.9146	0.9713	0.9576	0.8926	0.9409	0.9630	0.9341	0.9100	0.9003	0.9347
WNNM [40]	0.9651	0.9756	0.9765	0.9541	0.9610	0.9868	0.9674	0.9447	0.9592	0.9718	0.9535	0.9473	0.9369	0.9615
TSLRA [69]	0.9480	0.9743	0.9672	0.9536	0.9395	0.9803	0.9702	0.9261	0.9518	0.9758	0.9490	0.9361	0.9192	0.9532
SPG-SC	**0.9661**	**0.9845**	**0.9768**	**0.9621**	**0.9623**	**0.9906**	**0.9776**	**0.9465**	**0.9678**	**0.9814**	**0.9631**	**0.9495**	**0.9396**	**0.9668**
	Text Inlayed													
**Images**	**Mickey**	**Barbara**	**Butterfly**	**Fence**	**Haight**	**Leaves**	**Lena**	**Light**	**Lily**	**Pepper**	**Starfish**	**Tower**	**Zebra**	**Average**
BPFA [64]	0.9605	0.9658	0.9695	0.9555	0.9482	0.9721	0.9688	0.9530	0.9660	0.9782	0.9630	0.9530	0.9362	0.9608
IPPO [65]	0.9778	0.9841	0.9850	0.9764	0.9751	0.9892	0.9811	0.9675	**0.9771**	0.9881	0.9755	0.9685	0.9587	0.9772
ISD-SB [66]	0.9502	0.9336	0.9586	0.9195	0.9358	0.9501	0.9549	0.9077	0.9456	0.9721	0.9475	0.9345	0.9141	0.9403
JSM [7]	0.9727	0.9828	0.9836	0.9747	0.9729	0.9892	0.9792	0.9655	0.9715	0.9870	0.9732	0.9696	0.9535	0.9750
Aloha [60]	0.9530	0.9858	0.9653	0.9723	0.9492	0.9853	0.9755	0.9541	0.9630	0.9803	0.9528	0.9550	0.9429	0.9642
NGS [67]	0.9527	0.9633	0.9759	0.9412	0.9523	0.9558	0.9654	0.9396	0.9551	0.9639	0.9633	0.9465	0.9337	0.9545
BKSVD [68]	0.9474	0.9639	0.9463	0.9479	0.9423	0.9551	0.9584	0.9413	0.9524	0.9610	0.9479	0.9413	0.9359	0.9493
WNNM [40]	**0.9784**	0.9866	0.9865	0.9789	**0.9785**	0.9902	0.9806	**0.9714**	0.9769	0.9876	0.9762	0.9727	0.9604	0.9788
TSLRA [69]	0.9690	0.9829	0.9801	0.9725	0.9691	0.9843	0.9695	0.9621	0.9696	0.9832	0.9697	0.9633	0.9494	0.9711
SPG-SC	0.9779	**0.9878**	**0.9869**	**0.9797**	0.9784	**0.9908**	**0.9819**	0.9708	0.9765	**0.9884**	**0.9778**	**0.9728**	**0.9607**	**0.9793**

**Table 3:** PSNR (dB) comparison of BM3D [70], L0-ABS [71], ASDS [59], EPLL [72], NCSR [6], JSM [7], L2-r-L0 [73], WNNM [40], NLNCDR [74] and SPG-SC for image deblurring.

	9 × 9 Uniform Kernel, $\sigma_{n}=\sqrt{2}$														
**Images**	**Barbara**	**Bear**	**Fence**	**Lake**	**Lena**	**Lily**	**Flowers**	**Nanna**	**Corn**	**Agaric**	**Monk**	**Zebra**	**Man**	**Fireman**	**Average**
BM3D [70]	26.89	30.49	28.94	27.32	30.35	28.58	28.54	26.42	26.75	29.02	34.33	23.68	27.31	26.53	28.22
	0.7814	0.8074	0.8325	0.8230	0.8563	0.8119	0.8022	0.8001	0.8406	0.7695	0.8979	0.7561	0.7331	0.7435	0.8040
L0-ABS [71]	25.57	30.84	27.41	27.33	30.15	28.05	28.42	25.99	26.13	28.74	34.46	22.54	27.15	26.47	27.80
	0.7344	0.8246	0.7990	0.8289	0.8597	0.8004	0.7999	0.7925	0.8201	0.7629	0.9033	0.7353	0.7290	0.7490	0.7956
ASDS [59]	26.86	31.27	29.48	27.88	31.22	29.21	29.10	27.01	27.31	29.52	35.38	24.17	27.78	27.32	28.82
	0.7938	0.8333	0.8468	0.8344	0.8795	0.8290	0.8159	0.8261	0.8525	0.7954	0.9185	0.7844	0.7682	0.7801	0.8256
EPLL [72]	23.64	28.84	25.69	25.09	28.10	27.04	26.33	24.04	24.54	28.05	33.34	22.46	25.53	25.15	26.27
	0.7308	0.8250	0.7917	0.8287	0.8634	0.7981	0.8006	0.7961	0.8169	0.7585	0.9139	0.7364	0.7193	0.7423	0.7944
NCSR [6]	27.10	31.14	29.84	28.12	31.27	29.39	29.29	**27.07**	27.89	29.56	35.04	**24.64**	**27.91**	**27.40**	28.98
	0.7988	0.8264	0.8569	0.8472	0.8760	0.8393	0.8276	0.8286	0.8699	0.7980	0.9028	0.7984	0.7747	0.7857	0.8307
JSM [7]	25.72	28.32	27.26	26.22	28.05	26.97	27.15	25.47	25.69	27.42	29.99	23.32	26.36	25.71	26.69
	0.6953	0.6612	0.7456	0.7000	0.6953	0.6924	0.6524	0.7179	0.7751	0.6652	0.6698	0.7055	0.6589	0.6807	0.6940
L2-r-L0 [73]	26.07	31.10	27.92	27.88	30.44	28.47	28.73	26.52	27.00	29.08	35.04	23.35	27.47	26.77	28.27
	0.7610	0.8324	0.8167	0.8457	0.8712	0.8155	0.8125	0.8145	0.8479	0.7815	0.9185	0.7642	0.7468	0.7626	0.8136
WNNM [40]	27.23	31.33	30.16	28.17	31.35	29.25	29.21	26.86	**28.22**	29.52	35.53	24.33	27.68	27.23	29.01
	0.8065	0.8389	0.8570	0.8571	0.8898	0.8406	0.8336	0.8277	**0.8825**	0.7973	0.9257	0.7846	0.7602	0.7813	0.8345
NLNCDR [74]	26.22	30.75	28.23	27.58	30.44	28.69	28.73	26.59	26.68	29.16	33.73	23.36	27.65	27.02	28.20
	0.7552	0.8005	0.8181	0.8087	0.8380	0.8039	0.7889	0.8026	0.8300	0.7715	0.8581	0.7548	0.7512	0.7647	0.7962
SPG-SC	**27.51**	**31.37**	**30.12**	**28.21**	**31.42**	**29.40**	**29.35**	27.02	27.96	**29.61**	**35.80**	24.60	27.90	27.24	**29.11**
	**0.8187**	**0.8408**	0.8619	**0.8592**	**0.8914**	**0.8476**	**0.8431**	**0.8357**	0.8775	**0.8074**	**0.9287**	**0.7990**	**0.7796**	**0.7888**	**0.8414**
	Gaussian Kernel: fspecial(‘gaussian’, 25, 1.6), $\sigma_{n}=\sqrt{2}$														
**Images**	**Barbara**	**Bear**	**Fence**	**Lake**	**Lena**	**Lily**	**Flowers**	**Nanna**	**Corn**	**Agaric**	**Monk**	**Zebra**	**Man**	**Fireman**	**Average**
BM3D [70]	25.77	31.99	27.31	29.17	32.24	30.41	29.84	27.92	28.91	30.34	36.91	24.64	28.00	27.80	29.37
	**0.7987**	0.8618	0.7978	0.8836	0.9028	0.8701	0.8592	0.8652	0.8970	0.8368	0.9337	0.8127	0.7733	0.8138	0.8505
L0-ABS [71]	23.86	32.03	26.07	29.06	32.15	30.54	29.61	27.45	28.75	30.07	37.38	24.01	27.69	27.56	29.02
	0.7151	0.8776	0.7747	0.8934	0.9115	0.8801	0.8612	0.8660	0.9067	0.8392	0.9474	0.8055	0.7785	0.8236	0.8486
ASDS [59]	25.33	31.60	26.96	29.01	31.91	30.28	29.63	27.96	29.15	30.06	35.10	24.71	27.94	27.87	29.11
	0.7586	0.8246	0.7710	0.8450	0.8679	0.8368	0.8102	0.8460	0.8863	0.8113	0.8744	0.7997	0.7550	0.8042	0.8208
EPLL [72]	22.09	27.67	23.87	22.61	28.01	26.67	25.09	23.82	23.89	27.69	34.37	22.58	24.81	23.72	25.49
	0.6914	0.8570	0.7517	0.8562	0.8976	0.8519	0.8389	0.8322	0.8623	0.8118	0.9428	0.7813	0.7487	0.7854	0.8221
NCSR [6]	25.93	32.24	27.41	29.46	32.65	30.81	30.20	28.22	29.69	30.56	36.92	**25.05**	28.25	28.15	29.68
	0.7854	0.8621	0.8051	0.8865	0.9036	0.8773	0.8616	0.8681	0.9079	0.8466	0.9257	**0.8279**	0.7902	0.8294	0.8555
JSM [7]	25.89	31.31	27.08	28.97	31.48	30.03	29.52	27.84	29.01	29.96	34.43	24.66	27.88	27.76	28.99
	0.7592	0.8135	0.7719	0.8461	0.8487	0.8343	0.8082	0.8399	0.8861	0.8087	0.8481	0.8008	0.7543	0.8051	0.8161
L2-r-L0 [73]	24.18	32.44	26.50	29.51	32.53	30.53	29.97	27.97	29.40	30.30	37.78	24.23	28.12	27.87	29.38
	0.7301	0.8802	0.7850	0.8996	0.9176	0.8813	0.8656	0.8707	0.9111	0.8458	0.9543	0.8126	0.7843	0.8244	0.8545
WNNM [40]	25.51	**32.62**	27.46	29.65	33.00	30.90	30.17	28.16	**29.93**	30.68	**38.10**	24.53	28.24	28.14	29.79
	0.7669	**0.8837**	0.8061	**0.9017**	**0.9239**	**0.8886**	0.8741	0.8764	**0.9193**	0.8570	**0.9559**	0.8183	0.7897	0.8336	0.8639
NLNCDR [74]	24.43	31.19	26.67	28.99	31.42	30.17	29.47	27.71	28.88	29.92	34.32	24.46	27.84	27.81	28.81
	0.7295	0.8139	0.7654	0.8490	0.8515	0.8401	0.8132	0.8419	0.8881	0.8081	0.8502	0.7959	0.7587	0.8063	0.8151
SPG-SC	**26.08**	32.59	**27.50**	**29.67**	**33.03**	**30.91**	**30.27**	**28.25**	29.85	**30.84**	37.98	24.77	**28.31**	**28.20**	**29.88**
	0.7894	0.8804	**0.8104**	0.9002	0.9213	0.8880	**0.8751**	**0.8779**	0.9173	**0.8603**	0.9516	0.8261	**0.7955**	**0.8355**	**0.8664**

## Data Availability

Not applicable.
